# Role of Hypoxia in the Interferon Response

**DOI:** 10.3389/fimmu.2022.821816

**Published:** 2022-02-18

**Authors:** Esther Arnaiz, Adrian L. Harris

**Affiliations:** ^1^ Department of Oncology, University of Oxford, Oxford, United Kingdom; ^2^ Cambridge Institute for Therapeutic Immunology & Infectious Disease, Jeffrey Cheah Biomedical Centre, Cambridge, United Kingdom

**Keywords:** hypoxia, IFN, cancer, therapy, type I IFN

## Abstract

In solid tumors, as the tumor grows and the disease progresses, hypoxic regions are often generated, but in contrast to most normal cells which cannot survive under these conditions, tumour cells adapt to hypoxia by HIF-driven mechanisms. Hypoxia can further promote cancer development by generating an immunosuppressive environment within the tumour mass, which allows tumour cells to escape the immune system recognition. This is achieved by recruiting immunosuppressive cells and by upregulating molecules which block immune cell activation. Hypoxia can also confer resistance to antitumor therapies by inducing the expression of membrane proteins that increase drug efflux or by inhibiting the apoptosis of treated cells. In addition, tumor cells require an active interferon (IFN) signalling pathway for the success of many anticancer therapies, such as radiotherapy or chemotherapy. Therefore, hypoxic effects on this pathway needs to be addressed for a successful treatment.

## Introduction

As oxygen cannot diffuse more than 200μm from the closest capillary, poorly vascularized tumors or tumors that grow quick suffer from hypoxia and nutrient starvation. Hypoxia is linked to the hallmarks of cancer as it promotes tumor survival by inducing the switch to glycolytic metabolism, enhancing resistance to apoptosis and promoting escape from the immune system attack, among others (1). Hypoxia generates an immunosuppressive environment by avoiding the establishment and activity of immune effector cells and also by attracting immunosuppressive cells.

In addition, hypoxia is also involved in the regulation of specific cell signalling pathways required for the success of several anticancer therapies, such as the interferon pathway. Therefore, a better understanding of this pathway is necessary for therapy purposes.

## Interferons

Interferons (IFNs) comprise a family of cytokines first described in 1957 (2). The name was originally due to their ability to inhibit viral replication within cells. However, IFNs mediate a broad range of processes, not just antiviral action (3).

**Figure 1:**
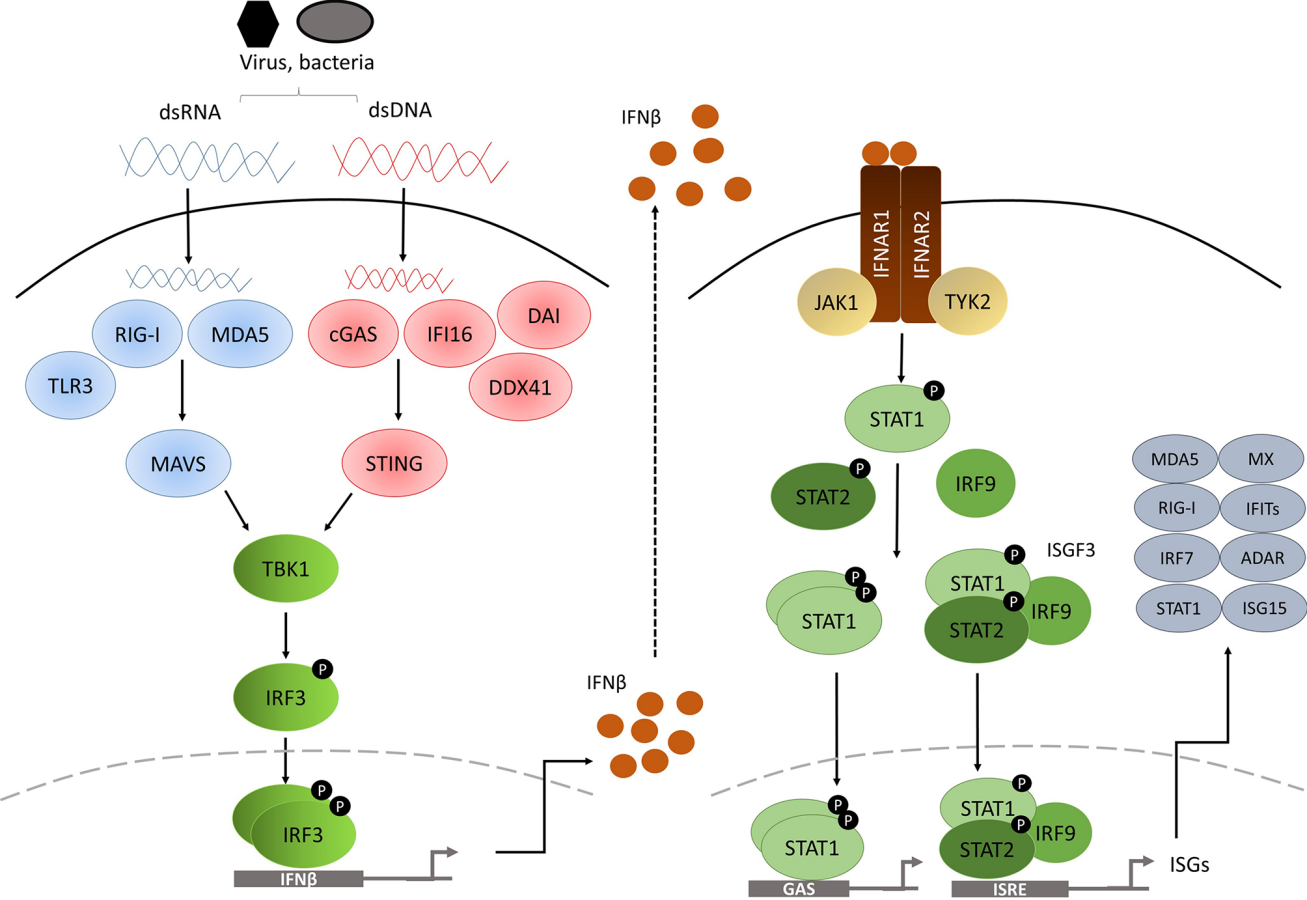
Diagram of the type I IFN pathway [modified from (8)].

Based on the receptor through which they signal, IFNs are classified as one of three types: type I, type II and type III (4). In humans and mice, there are 17 different type I IFNs including 13 IFNα subtypes, IFNβ, IFNϵ, IFNκ and IFNω. In contrast, type II IFNs consist of a single member, IFNγ, and type III IFNs consists of 4 IFNλ subtypes. Nevertheless, all IFNs are involved in the innate response against pathogenic infection upon detection of microbial products, known as pathogen-associated molecular patterns (PAMPs), such as genetic material, viral glycoproteins or bacterial lipopolysaccharides (LPS), *via* pattern-recognition receptors (PRRs) (5–7), leading to production and secretion of IFNs to activate an immune response against the infection in multiple cell types. These cytokines can exert direct antimicrobial response by inhibiting pathogen replication and inducing cell death of infected cells or can act in a paracrine manner in non-infected adjacent cells promoting them to produce an array of genes called interferon-stimulated genes (ISGs) to prevent pathogen spread (**Figure 1**) (9).

## The IFN Pathway

The PRRs are located in various cellular compartments and detect different components of the pathogen during the course of the infection, and they are classified into cytosolic PRRs or transmembrane PRRs. Cytosolic PRRs include the ubiquitously-expressed retinoic acid-inducible gene I (RIG-I) and melanoma differentiation-associated gene 5 (MDA5) for double-strand RNA (dsRNA) sensing. The cyclic GMP-AMP (cGAMP) synthase (cGAS), DExD/Hbox helicase 41 (DDX41), DNA-dependent activator of IFN-regulatory factors (DAI, also known as ZBP1) and interferon-γ-inducible factor 16 (IFI16) provide for double-strand DNA (dsDNA) sensing. In contrast, transmembrane PRRs comprised of Toll-like receptor (TLR) family members are limited to specialized cells such as macrophages and DC.

### Cytosolic PRRs

RIG-I and MDA5 are both caspase recruitment domain (CARD)-containing RNA helicases able to recognize dsRNA. RIG-I is activated by short dsRNA molecules, whereas MDA5 recognizes long (>2kb) dsRNAs. Both dsRNA forms can arise from the genetic material of a dsRNA virus, or alternatively can be generated by single-strand RNA (ssRNA) viral replication in the cell (10, 11). Interaction of either of the PRRs with viral RNA induces unwinding of the dsRNA and conformational changes to RIG-I and MDA5 that expose the CARD-containing domains, which in turn interact with the CARD-containing domain of IFNβ promoter stimulator-1 (IPS-1) (12), also known as mitochondrial antiviral-signalling protein (MAVS), which is located in the mitochondrial membrane (**Figure 1**). This leads to TANK binding kinase 1 (TBK1) activation *via* TRAF3 and to inducible IκB kinase (Iκκ) activation *via* TRAF6. TBK1 is essential for the phosphorylation of IRF3 and IRF7 (13, 14), whereas the contribution of Iκκ to the cytosolic pathway is minor, although it also phosphorylates IRF3 and IRF7 (15). After being phosphorylated, IRF3 and IRF7 form homo and heterodimers which will induce the expression of chemokines, inflammatory cytokines and IFNβ after their translocation to the nucleus (16–18).

Importantly, various other receptors have been described to be involved in dsDNA sensing in the cytosol. For example, the dsDNA sensor cGAS undergoes conformational changes after binding to dsDNA, inducing its catalytic activity to generate the second messenger cGAMP, which will bind to and activate the stimulator of interferon genes (STING) adaptor protein that resides in the endoplasmic reticulum of the cell (19, 20). On the other hand, once dsDNA is bound, DDX41 (21), IFI16 (22) and DAI (23) can directly bind to STING, which in turn translocates to signalling compartments *via* the Golgi apparatus (24), where it associates with TBK1 and activates IRF3 by phosphorylation (25). NF-κB is also activated by dsDNA sensing, and appears to collaborate with IRF3 to induce type I IFN gene expression (26).

However, it has recently been described that STING exerts antiviral response activities independent of type I IFNs. Two independent studies showed that mice harbouring serine 365-to-alanine (S365A) mutation in the C-terminal tail (CTT) of STING were protected against *Herpes Simplex Virus* (HSV)-1 infection, despite lacking a functional STING-IFN response (27, 28). This suggests that STING can control pathogenic infection through IFN-independent mechanisms.

### Transmembrane PRRs

TLR family members offer a broad coverage to detect almost every pathogen, as they can recognize many different forms of genetic material and also extracellular components, leading to IFN pathway activation. For instance, TLR3 recognizes dsRNA, whilst TLR7 and TLR8 detect ssRNA (29) and TLR9 identifies unmethylated CpG islands (30), in addition to TLR4 that binds to LPS present in the membrane of Gram-negative bacteria (31). TLRs that bind genetic material are located in the endosomal compartment, whereas TLR4 signals from the cell surface, but also from the endosomes. Upon recognition of foreign material, TLRs recruit adaptor proteins through their Toll-IL-1 receptor (TIR) domain, such as myeloid differentiation primary response gene 88 (MyD88), TIR domain-containing adaptor protein (TIRAP), TIR domain containing adaptor-inducing IFN (TRIF) and the TRIF-related adaptor molecule (TRAM) (32). Signalling can be categorized into two pathways: MyD88 pathway (signals coming from TRL4, TRL7, TLR8 and TLR9) or TRIF pathway (after TLR3 and TLR4 activation) (33).

After the activation of their specific adaptors, cytosolic and membrane PRR pathways converge to promote the phosphorylation of IRF3 and IRF7, which will dimerize and translocate to the nucleus to induce the expression of type I IFN genes (15).

## Type I IFN Signalling

Type I IFNs initiate their signalling *via* binding to IFNα receptor (IFNAR), expressed on the surface of all nucleated cells. All type I IFNs bind to this heterodimeric receptor formed by the subunits IFNAR1 and IFNAR2 (4). IFNAR1 is considered the ‘signal transduction’ chain, whereas IFNAR2 is more likely to be the high affinity binding chain of the receptor. On engagement, the intracellular domains of IFNAR1 and IFNAR2 move together, as do their associated signalling adaptors, tyrosine kinase 2 (TKY2) and Janus kinase 1 (JAK1) (4). TKY2 and JAK1 are then activated by reciprocal phosphorylation (34), and they phosphorylate tyrosine residues on the receptor that will act as docking sites for the signal transducer and activator of transcription (STAT) proteins (35). Recruited STAT proteins are phosphorylated by JAK and other kinases, leading to their dissociation from the receptor, activation and dimerization (35).

The canonical signalling cascade involves STAT1 and STAT2. These two transcription factors form a ternary complex called interferon-stimulated gene factor 3 (ISGF3) with interferon-regulatory factor 9 (IRF9). ISGF3 complex translocate to the nucleus, where it binds to a DNA sequence motif known as interferon-stimulated response elements (ISREs) (35). In addition to ISGF3, type I IFNs also promote the formation of STAT1 and STAT3 homo or heterodimers. In this case, the dimers bind to a different sequence called gamma-activated sequence (GAS) (36). STAT2/IRF9 complex, in the absence of STAT1, can also be generated (37). Moreover, type I IFNs can activate downstream signalling through STAT-independent pathways such as p38 and extracellular signal regulated kinase (ERK) (3). All these pathways will finally activate the transcription of ISGs involved in different mechanisms to restrain pathogens.

Most ISG-encoded proteins contribute to immune-stimulatory and antiviral effects. Moreover, PRRs, JAKs and STATs are also ISGs, therefore reinforcing IFN signalling. Some ISGs, such as IFIT proteins, IRF9, MX1 or MX2 have direct antiviral activity (38). While various ISGs function as chemo-attractants to lymphocytes and monocytes, others have pro-apoptotic effects, such as TRAILS, Fas/FasL or ISG12.

## Acute vs. Chronic IFN Response

Cells have a tight regulation of IFN-induced immune responses. Whereas an intact IFN signalling is required for acute antiviral activity, it needs to be weakened to avoid tissue damage. It is therefore not surprising that cellular responses to IFNs vary during the course of the infection. Cells can modulate the magnitude of the response firstly at receptor level, by generating a truncated form of the receptor incapable of signalling (39) or by ubiquitinating, endocytosing and later degrading the receptor in the lysosomes (40). The level of endocytosis and receptor degradation correlates with the degree of signal (41). High IFNAR1 levels in the cell membrane and high and tight binding of type I IFNs to the receptor will strongly promote IFNAR1 degradation, whereas low type I IFN concentration or low affinity IFN binding will not have such a marked effect on the receptor levels.

Furthermore, the signal transduction TYK2/JAK1-STAT axis is a potential target for some dephosphorylation proteins known as suppressors of cytokine signalling (SOCS) (42, 43). Unrestricted type I IFN signalling, due to a lack of SOCS1, induced inflammation and was potentially lethal in mice (44). Moreover, epigenetic modification of JAK1 (45) or STAT ubiquitination (46) makes cells unresponsive to IFNα.

Finally, several ISGs control the magnitude of the IFN response. USP18 is involved in the negative regulation of the IFN signalling by decreasing the stability of IFN-IFNAR binding (47) or removing ISG15 from its substrates (48). IFIT1 overexpression resulted in the inhibition of IRF3 activation, NF-κB activation and IFNβ production (49). While the interferon-inducible isoform of Adenosine Deaminase Acting on RNA (ADAR-p150) edits RNA duplexes in self mRNAs by deaminating adenosines into inosines to prevent aberrant activation of the IFN pathway (50).

However, if the IFN signalling persists, it acquires immunosuppressive features. This chronic IFN response is detected in various autoimmune diseases, such as systemic lupus erythematosus (SLE), Sjogren’s syndrome, systemic sclerosis or rheumatoid arthritis, as well as in patients with chronic infections such as hepatitis C virus (HCV) and HIV (51). Type I IFN pathogenic role in autoimmune diseases is thought to be due to the promotion of antigen presentation, to enhance lymphocyte responses and to the induction of chemokines. Besides, some immune cells express high levels of the signal transducer molecules (52).

On the other hand, IFNs can also have a protective role in autoimmune diseases by suppressing the generation and activity of inflammatory cytokines, inhibiting pathogenic cell proliferation or limiting T cell responses (53). In addition, type I IFNs can also induce the expression of suppressive molecules such as IL-10 or the immune checkpoint inhibitor molecule programmed cell death ligand 1 (PD-L1), which if exceed the pro-inflammatory cytokine levels, will abrogate the clearance of the viral infection (54, 55). Therefore, under chronic viral or bacterial infection, type I IFN signalling acquires an immunosuppressive role, probably to limit the toxicity caused by persistent infection. As type I IFNs can exert both deleterious and protective effects in autoimmune diseases, it is likely that IFN responses change over the course of the infection or disease, activating or suppressing the immune response. This is critical in the consideration of the role of IFNs in cancer.

## IFNs and Cancer

Type I IFNs can be produced in the absence of infection, such as in the presence of DNA from apoptotic cells (56), non-removed nucleic acids (57) and autoimmune disease antibodies (58).

In 1909 Paul Ehrlich first proposed that the immune system could recognize and eliminate tumor cells (59). However, this was not widely accepted and given the name of ‘cancer immunosurveillance’ until the late 50s (60, 61). The first experiments to test this hypothesis in the 1970s were not successful as they were carried out on mice that were only partially immunodeficient and the knowledge about the immune system was limited (62). In the 1990s, endogenous IFNγ was demonstrated to protect the host against transplanted, chemically induced and spontaneous tumor growth (63–65). Experiments with mice lacking the recombinase activating gene (RAG)-2, and thus lacking lymphocytes T, B and natural killer (NK) T cells, further demonstrated that tumor development in mice was controlled by the immune system, as RAG-2 deficient mice were more sensitive to MCA-induced sarcomas (66).

Nevertheless, it was noted that tumor formation still occurs in individuals that are immunocompetent, suggesting that tumor cells are able to escape the immune system pressure (67). This idea arose from the observation that tumors from immunocompetent and immunodeficient hosts have different immunogenic properties (68). Tumors developed in mice with an intact immune system formed progressively growing tumors when transplanted into immunocompetent recipients, whereas tumors originated in the absence of an immune system were rejected when transplanted into immunocompetent hosts, but grew in immunodeficient hosts (66, 69). This suggested that the immune system performs an ‘editing’ process on tumor cells. It can eliminate highly immunogenic tumor cells, but less immunogenic cells can escape the deletion phase and generate tumors poorly recognized by the immune system (68). Therefore, the immune system provides protection against tumor development, but it can also promote tumor growth by selecting tumor cells with low immunogenicity. This dual effect made to refine the cancer immunosurveillance hypothesis, which was further renamed as ‘cancer immunoediting’ (66, 67).

### Cancer Immunoediting

The editing process that the immune system performs in tumors can be divided into three phases: elimination, equilibrium and escape (67). Tumor cells and immune cells interact during the whole process of tumor formation and these interactions are mediated by endogenously produced type I and type II IFNs.

#### Elimination Phase

The elimination phase, also termed protection, is the first step in the cancer immunoediting. If successfully performed, it will eradicate the developing tumor and the immunoediting process will not progress to subsequent phases (68).

In this phase, both the innate and adaptive immune system recognize cancer cells and kill them. The antitumor immune response starts when cells of the innate immune system detect that a tumor is growing. As a result, the disruption of the tissue surrounding the tumor generates pro-inflammatory molecules that together with chemokines secreted by tumor cells act as danger signals for the immune system (70). Once immune cells reach the tumor mass, they need to distinguish between normal self-cells and transformed self-cells. This is achieved by recognizing tumor-cell-expressed ligands for the NK cell receptor Natural killer group 2 member D (NKG2D), which are induced by genotoxic stress, the generated inflammation or the transformation process itself (71), or tumor-specific antigens presented by antigen presenting cells (72).

This recognition process leads to chemokine and cytokine production at the tumor site. Released IFNs induce the production of more chemokines, which will attract more immune cells to the tumor and will in turn produce more cytokines, and therefore the signal will be amplified (68). The massive release of molecules will activate different processes, including antiproliferative (73), pro-apoptotic (74) and angiostatic (75) processes to kill tumor cells. The main two cell populations recruited by type I IFNs are NK cells and cytotoxic T cells (CTLs). As tumor cell death generates tumor-cell antigens, the adaptive immune system will start to take part in the immunoediting process.

#### Equilibrium Phase

This is a subclinical phase in which the tumor persists, but it is prevented from expanding due to the presence of the immune system. It is probably the longest phase, and can even take years to occur in humans (76). The immune system is able to contain any tumor cell variant that has not been killed in the previous phase, but it is not able to completely remove the tumor. Furthermore, during this phase, new tumor cell variants arise that display reduced immunogenicity (i.e. more resistant to the immune attack) (68).

#### Escape Phase

The escape phase is the tumor progression phase. It is the result of immune exhaustion or inhibition, or the consequence of the emerged new cell population’s ability to bypass the immune pressure. Tumor cells can impede an antitumor immune response either through the production of immunosuppressive cytokines (TGFβ and IL-10) or *via* T cells with immunosuppressive activities (Treg cells) (68). New tumor cells might have also been submitted to alterations that make them unrecognizable by immune cells or that allow them to avoid immune destruction (64). These poorly immunogenic cells will be able to grow and generate a clinically detectable tumor (68, 77).

Apart from the host’s ability to produce IFNs, tumor cell responsiveness to IFNs is also important for a successful antitumor immune response. Tumor cells insensitive to IFNγ due to a mutant IFNγ receptor (IFNGR1), grew more aggressively when transplanted into a wild type host (63), but when those cells were made responsive again by complementation with a wild type IFNGR1, they became highly immunogenic and failed to form tumors in the recipients (64). Similarly, it was observed that several highly immunogenic and poorly tumorigenic sarcomas from RAG-2 deficient mice were converted into poorly immunogenic, and thus highly tumorigenic, when rendered insensitive to IFNγ (66). These observations suggest that IFNγ production both by tumor cells and tumor host is relevant for an adequate immunosurveillance.

Although type I IFNs were thought to be mainly antiviral agents, their importance as immunomodulators has become clear. In the 1960s, it was demonstrated that tumor-bearing mice had a better outcome when treated with type I IFNs (78, 79). Although these studies showed the antitumor potential of type I IFNs, it was not until 1981 when the role of endogenously produced type I IFNs was assessed. When mice challenged with tumor cells were treated with serum containing anti-IFN specific antibodies, they developed bigger tumors and had decreased survival comparing to mice treated with control serum (80, 81). In a similar way, endogenously produced type I IFN was required to protect the host against transplanted and primary carcinogen-induced tumor growth (82). This study also showed that in contrast to IFNγ, type I IFNs do not require tumor cell responsiveness to exert the antitumor immune response. In spite of being insensitive to IFNα/β, 4 of 11 IFNAR1-deficient MCA-induced sarcomas were rejected when transplanted into wild type recipients (82). Moreover, some IFNAR1-deficient sarcomas that grew in wild type recipients were not converted into highly immunogenic, non-growing tumors when IFNAR1 expression was restored. Besides, sarcoma cells from mice lacking IFNAR1 and IFNGR1 were only rejected when sensitivity to IFNγ was restored, not to type I IFN. On the other hand, IFNα/β sensitivity in hematopoietic cells of the host was required for an antitumor immune response (82).

### IFNs and Anticancer Therapies

Type I IFNs are key components in the success of many of the current anticancer modalities including radiotherapy, chemotherapy, immunotherapy and oncolytic viruses (83), through promotion of direct (tumor cell inhibition) and indirect (antitumor immune response) effects. Indirect effects, recently termed ‘immunogenic cell death’ (ICD), arise as a consequence of the release of nucleic acids or proteins from dying tumor cells that can activate immune cells (84).

Regarding radiotherapy, type I IFN production by myeloid cells and IFN detection by tumor cells after radiotherapy was essential to eradicate the tumor in a mouse melanoma model, as mice lacking IFNAR1 were unresponsive to IFNβ (85). Similarly, radiation-induced type I IFNs recruited lymphocytes at the tumor site and exogenously administrated IFNα enhanced radiotherapy efficacy (86). Furthermore, radiation-mediated antitumor immunity in immunogenic tumors requires a functional cytosolic DNA-sensing pathway in dendritic cells (DC), which subsequently triggers a type I IFN signalling response in those cells essential for CTLs activation (87).

As with radiotherapy, the anticancer effects of chemotherapy, which were long thought to be mediated only by direct tumor cell killing, are now realized to be due in part to the induction of ICD, thereby involving the immune system in the process. Injection of mice with tumor cells pretreated with doxorubicin prevented *in vivo* tumor growth in immune competent mice only, although the mechanism was not elucidated at that time (88). Chemotherapeutic agents used in the clinic such as anthracyclines (e.g. doxorubicin, epirubicin, mitoxantrone), radiomimetics (bleomycin) or platinum analogues (oxaliplatin) induce ICD, *via* IFN signalling (89). For successful treatment, anthracyclines must be able to induce the release of type I IFNs in both tumor and immune cells so that an antitumor adaptive immune response against tumor-cell antigens can be activated (90). This study also showed that IFNAR1 expression in cancer cells is important for the activity of anthracyclines, as administration of an IFNAR1 neutralizing antibody abolished the therapeutic activity of the drugs. Moreover, a type I IFN-related gene signature in patients with breast carcinoma treated with anthracycline-based chemotherapy was able to predict clinical responses to the treatment (90). As an example, chemotherapy upregulated the levels of MX1 and its high levels were associated with better overall survival in those patients.

Immunotherapy has become a major treatment modality in the last few years, because of the development of immune checkpoint inhibitors (ICIs). By blocking immune checkpoints, the level of T cell activation can be controlled, thus avoiding excessive inflammation and promoting self-tolerance (91). The two main targets are CTLA-4 and PD-1 expressed by activated T cells, and PD-L1 in tumors and inflammatory cells. CTLA-4 controls T cell activation by competing with its homologous T cell costimulatory receptor (CD28) in the binding to their ligands. Both molecules bind to CD80 and CD86 (also called B7-1 and B7-2) expressed by antigen-presenting cells, but CTLA-4 transmits an inhibitory signal to T cells, whereas CD28 transmits a stimulatory signal. Thus full activation of T cells requires binding of CD28 to CD80 and CD86, but in tumors, CTLA-4 is frequently upregulated, as a consequence of T cell exhaustion due to long time exposure to tumor cells (91). Similarly to CTLA-4, PD-1 regulates T cell activation by binding to PD-L1 and PD-L2 expressed in infiltrating inflammatory cells and tumor cells. Cancer cells overexpress PD-L1 which blocks T cell activation and finally dampens the antitumor immune response (92). In this regard, antibodies against immune checkpoint modulators restore the effector T cell functions and the antitumor immune response.

Despite existing approved monoclonal antibodies targeting CTLA-4, PD-1 and PD-L1 for the treatment of lung cancer, RCC and melanoma, amongst many others, the percentage of patients responding remains modest, although for those responding it is of major importance (93, 94). These therapies rely on the increase of immune responses, and given the importance of type I IFNs in the maturation, survival and activation of most immune cells (82), it is likely that a functional IFN pathway is necessary to achieve success with these therapies. Consistent with this idea, Zaretsky et al. demonstrated that melanoma patients who relapsed after treatment with the PD-1 inhibitor pembrolizumab, had loss-of-function mutations in genes encoding JAK1 and JAK2 (95). Similarly, defects in IFNγ signalling led to resistance to PD-1 and CTLA-4 blockage (96), and lung tumors resistant to anti-PD-1 therapy presented mutations in IFNAR2 and IFNγ signalling pathway (97). In addition, a transcriptome study analyzing advanced melanoma patients treated with nivolumab (anti-PD-1) alone or combined with ipilimumab (anti-CTLA-4) showed high T cell infiltration and IFNγ signalling signatures in patients clinically responding to the therapy. In addition, *in vitro* studies showed that IFNγ exposure led to similar transcriptome responses unless IFNGR was altered (98). This study further supports that a functional IFN signalling and a potent antitumor T cell response are critical for the success of immune checkpoint therapies.

However, another study reported that IFN signalling in cancer cells and immune cells had to oppose each other to stablish a regulatory relationship (99). They showed that whereas inhibition of tumor IFNγ signalling led to reduced ISG expression in tumor cells, it increased ISG levels in immune cells by enhancing IFNγ production by exhausted T cells.

Oncolytic virus therapy is based on the ability of modified virus to specifically target tumor cells, whilst not affecting healthy cells. In contrast to other therapies, oncolytic virus therapy requires a defective IFN signalling to succeed, since viruses are susceptible to IFN-mediated antiviral activity. HSV could destroy murine breast tumor cells unable to produce and respond to IFNs (100). In the same way, cancer cell sensitivity to *Vesicular stomatitis virus* (VSV) induced cell death was increased after knocking down or blocking IFNAR (101), IRF5 and IRF7 (102), thus providing evidence for the requirement of a defective IFN pathway.

On the other hand, the induction of type I IFNs during combined adoptive cell therapy and oncolytic virus therapy led to an autoimmune response, as blocking both IFNα and IFNβ completely abrogated the autoimmune side effect without affecting the antitumor efficacy of the treatment (103). This study showed that although a functional type I IFN is required for the success of many therapies, it is also important to control the magnitude of the response, as immunotherapy-induced type I IFN secretion can have a pathogenic role inducing autoimmune toxicity.

## Cancer Hypoxia

One of the main characteristics of solid tumors is hypoxia. During tumor growth, an oxygen concentration gradient is generated within the tumor mass, as oxygen can only diffuse 100-180µm from the closest capillary, whereby internal cells become oxygen deprived otherwise known as hypoxia. In response to the stress generated by oxygen deprivation, cells activate several adaptive responses to match oxygen supply with their energetic demands, most of them driven by the hypoxia-inducible transcription factors (HIFs). Although first identified as a regulator of erythropoietin (EPO) production (104), HIFs are now recognized as the key modulators of the hypoxic response. These transcription factors function as heterodimers formed by an oxygen sensitive α subunit (HIF1α, HIF2α and HIF3α) and a stably expressed β subunit (HIF1β), also named aryl hydrocarbon receptor nuclear translocator (ARNT) (105).

HIFα is tightly regulated by oxygen-independent (iron, α-ketoglutarate and ascorbate) and oxygen-dependent mechanisms. Under normal oxygen tension (i.e. normoxia), prolyl hydroxylase domain enzymes (PHDs) hydroxylate HIFα subunits at conserved proline residues, allowing the binding of the von Hippel Lindau (VHL) E3 ubiquitin ligase complex which will polyubiquitinate HIFα targeting it for proteasomal degradation (106, 107). HIFα can also be hydroxylated in an asparagine residue by Factor Inhibiting HIF (FIH), which will avoid HIF interaction with the coactivators required for the transactivation of the target genes (108, 109). Under hypoxia however, PHDs and FIH are not active, HIFα is stabilized and can translocate to the nucleus and dimerize with HIF1β. This heterodimer will bind to specific DNA domains called ‘hypoxia response elements’ (HRE) of target genes and will activate their expression. HIF activation in hypoxic cells promotes tumor progression, as they activate genes involved in cell proliferation, angiogenesis, apoptosis, metabolism, DNA damage response, ECM remodeling, cell migration and invasion and evasion of the immune system.

## Hypoxia And The Immune System

Hypoxia generates an immunosuppressive microenvironment within the tumor by impeding the homing of immune effector cells (T cells and NK cells), by blocking their activity and by recruiting immunosuppressive cells, such as myeloid-derived suppressor cells (MDSC), tumor-associated macrophages (TAM) and Treg cells.

MDSC induce T cell anergy, block CD8^+^ T cell activity and promote Treg cell proliferation (110). Hypoxia can further promote MDSC differentiation to TAM (111). On the other hand, TAM infiltrating hypoxic tumor regions are associated with an M2 immune evasive phenotype (112), as they suppress T cell function (113). Moreover, it is well known that hypoxia significantly reduces lymphocyte proliferation directly (114), and it was observed *in vivo* that when hypoxic areas were reduced in tumors, immune cell infiltration increased (115, 116). In addition, hypoxia can also inhibit T cell proliferation by upregulating Treg cells (117).

Apart from the specific effect of hypoxia on immune cells, hypoxic cells can evade innate immune system by expressing specific molecules, such as the ‘don’t eat me signal’ CD47 molecule (118), or the immune checkpoint inhibitor molecules PD-L1 and CTLA-4 (119, 120), which will bind to macrophages and effector T cells and block their activity. Furthermore, under hypoxic conditions, PD-L1 expression is also upregulated in MDSC (121). Similarly, major histocompatibility complex class I chain related (MIC) molecules, one family of NKG2D receptor ligands are upregulated in many tumor cells (122). This would effectively lead to NK and T cell mediated immunosurveillance. However, hypoxic cancer cells secrete MIC ligands (soluble MIC) to downregulate and degrade NKG2D receptor on T cells and avoid immune activity (117).

As a consequence of the metabolic switch to glycolysis that tumor cells undergo under hypoxic conditions, lactic acid levels in the extracellular media increase considerably. Recent data highlights the role of lactate as a ‘signalling molecule’ promoting the escape of the immune surveillance of hypoxic tumor cells (123). Lactate can attenuate the cytotoxic activity of CTLs (124) and NK cells (125), and recruit MDSC to the tumor (125). Furthermore, glucose-deprived T cells, due to the high glucose uptake by cancer cells, have lower antitumor effector functions (126, 127).

On the other hand, hypoxia upregulates the expression of CD39 and CD73, enzymes involved adenosine metabolism, on tumor cells, as well as A2A adenosine receptor (A2AR) on immune cells (128). Extracellular adenosine signals through A2AR on immune cells and inhibits their activity (129). Additionally, Treg cells express high levels of CD39 and CD73 and they are able to produce and secrete adenosine, contributing to immune evasion (130).

Altogether this shows that hypoxia generates an immunosuppressive environment within the tumor mass, by recruiting immunosuppressive cells and by upregulating the expression of molecules involved in immune system evasion. In view of the role of IFNs in the regulation of these cells, the specific effects of hypoxia on IFN regulation and signalling are reviewed below.

## Hypoxic Regulation of the IFN Response

Regarding the type I IFN pathway, hypoxia induces damage to mitochondria and makes these organelles release damage associated molecular patterns (DAMPs), such as mitochondrial DNA (mtDNA), able to trigger an immune response (131). However, hypoxia downregulates the cytosolic dsDNA sensor cGAS, thus reducing STING activation and the consequent type I IFN response, *via* increasing the levels of miR-25 and miR-93 (132). This allows hypoxic tumor cells to evade the immune responses induced by DAMPs, and promotes the establishment of an immunosuppressive tumor microenvironment. In addition, hypoxia downregulated the levels of the dsRNA sensors RIG-I and MDA5, in addition to other members of the pathway, *via* a HIF-independent reduction in chromatin accessibility (8). Similarly, 2% hypoxia reduced RIG-I and MDA5 protein expression in malignant cells in a HIF1α-dependent manner, even after treating the cells with the type I IFN activator 5’-triphosphate RNA (3pRNA), whereas non-malignant or primary cells presented similar levels (133). Moreover, the high lactate levels found in hypoxic tumors inhibit type I IFN signalling *via* binding to MAVS adaptor protein (134). Besides, hypoxic inhibition of type I IFN production and less cellular responses to these cytokines in hypoxia cooperate to suppress immune responses under low oxygen conditions. The negative effect of tumor hypoxia on radiotherapy efficacy is partially due to hypoxic inhibition of IFNγ production and the impairment of hypoxic cells to response to IFNγ (135). Using murine tumor cells lines, Murthy et al. observed that hypoxia inhibited IFNγ-dependent gene expression in tumor cells, reducing immune cell infiltration. Furthermore, CD8^+^ T cells had reduced ability to proliferate and generate IFNγ under hypoxic conditions, but reoxygenation restored the cytokine-producing capacity of these cells.

Altogether these studies show that hypoxia is impairing the type I IFN response and therefore the effector immune cell responses in tumors. Moreover, IFNγ and IFNα (to a lesser extent) induced the transcription of prolyl hydroxylase 3 (PHD3), a HIF-inducible negative regulator of HIF transcription factors, in human endothelial cells (136). IFNγ induced PHD3 expression *via* JAK/STAT1 signalling independent of HIF1α. They also showed that pharmacological inhibition of PHDs suppressed the induction of IFNγ-dependent genes after IFNγ treatment.

On the other side, hypoxia and IFNγ synergistically work to fully activate macrophages infiltrating hypoxic tumor regions (137), *via* HIF1α and IFN regulatory factor-1 (IRF-1) interaction (138). Activated macrophages express the inducible NOS2 and produce NO, which will promote tumor cell apoptosis. In line with this result, *RIG-I* gene presents a HRE, and RIG-I expression was found to be upregulated in human muscle cells under hypoxic conditions (139) as well as in kidneys of hypoxic mice (140). Supporting the contribution of hypoxia to IFN signalling, HIF1α lacking mouse embryonic fibroblast expressed lower levels of IFNβ1 upon infection with the DNA virus murine gamma herpesvirus 68 (MHV68), whereas the levels of IFNα4 and IFNα6 remained unchanged (141). Similarly, transcript levels of the IFN-stimulated gene OAS1β were also reduced. This study supports that hypoxic regulation of the type I IFN pathway is not always dependent on HIF1α, as previously described (8). Another study showed that CD4+ T cell stimulation under hypoxic conditions augmented the secretion of effector cytokines, especially IFNγ, and that hypoxic effects on IFNγ secretion were not dependent on HIF1α deficiency (142). Furthermore, human mesenchymal stromal cells (MSCs) contain immunosuppressive characteristics which makes them promising candidates for treating immune disorders, however, they require specific signals to acquire this phenotype. Wobma et al. found that combination of IFNγ and hypoxia priming produces more immunosuppressive MSCs than when either cue is used alone. They showed that IFNγ induced the expression of several immunosuppressive proteins while hypoxia switched MSCs to glycolysis, causing the production of the T cell inhibitor lactate (143).

All these studies demonstrate that although there is a general negative effect of hypoxia on the IFN response, there are also conditions in which hypoxia has a positive effect, contributing to and enhancing the IFN signalling.

In regard to infectious diseases, it has been described that respiratory viruses such as severe acute respiratory syndrome coronavirus 2 (SARS-CoV-2) and influenza A encode antagonists of the IFN response (144). Recent clinical studies have shown that COVID-19 patients present low IFN levels and that this correlates with severe infection. It has been described that COVID-19 infected patients present hypoxic monocytes unable to produce type I IFNs after activation by the alarmin HMGB1, as HIF1α acted as a direct transcriptional repressor of IRF5 and IRF3 (145).

On the other hand, a study from 1993 showed that oxygen tension greatly affects the response to IFNα and IFNγ *in vitro*. Under hypoxic conditions, the cytopathogenicity of vesicular stomatitis virus (VSV) was reduced, whereas the antiviral effects of the IFNs were increased. These results suggested that hypoxia is a possible host defense system against viral infection, *via* potentiating the effects of the IFNs produced at the site of infection (146). Similar results were obtained more recently, showing that elevated HIF activity confers resistance to VSV mediated cytotoxicity, as inhibition of HIF increased cellular sensitivity to infection and CoCl_2_ treatment to mimic hypoxia promoted resistance (147). They also detected that HIF promoted *IFNβ* expression, among other antiviral genes, upon viral infection. These results suggest that in contrast to tumor hypoxia where the IFN signalling is suppressed, upon viral infection, hypoxic regions protect the host and help clearing up the infectious agent.

## Translational Impact

The aim of immunotherapy is to increase the number and functionality of antitumor effector cells, and ICIs are becoming the primary treatment modality in this regard. However, only a subset of tumor types responds to these treatments (93, 94), and this is mainly due to the tumor and the tumor microenvironment generated immunosuppression. As mentioned before, tumors possess different mechanisms to generate an immunosuppressive environment, such as recruiting immunosuppressive cells or express specific molecules, in addition to the well-described effect of hypoxia. Therapeutic approaches that modulate tumor hypoxia have been shown to improve immunotherapy response.

One of the strategies to improve T-cell function is to reverse tumor hypoxia by reoxygenation (148). Respiratory hyperoxia promoted effector T-cell infiltration into the tumor (115). In addition, inhibition of oxidative phosphorylation directly alleviated hypoxia and improved the effectiveness of anti-PD-1 therapy (149).

Another way of bypassing hypoxia-induced immunosuppression is using hypoxia-activated prodrugs (HAPs). HAPs are agents designed to selectively activate under hypoxic conditions by enzymatic reduction, generating an active compound able to kill hypoxic tumor cells. It was observed that the increased blood vessel density after evofosfamide treatment promoted tumor infiltration of T-cells and improved ICIs therapy (116, 150). Similarly, metabolism of CP-506 in hypoxic regions releases active metabolites which form DNA crosslinks leading, ultimately, to tumor cell death. It was observed that CP-506 synergies with ICIs (151), suggesting that combination of HAPs and ICIs is a good therapeutic approach to treat hypoxic tumors. Moreover, most HAPs generate DNA-damaging cytotoxic effects, but they can also be designed to release effectors against a range of molecular targets, such as tarloxotinib, a pan-HER inhibitor (152).

Similarly, there are some nanoparticle agents in preclinical development, both oxygen-carrying (153) and oxygen-generating (154), designed to increase tumor oxygenation. Zhou et al. established a two-stage oxygen delivery system to relieve tumor hypoxia. They found that perfluorotributylamine (PFTBA) nanoparticles were able to inhibit platelet activation leading to an increase in red blood cell infiltration and therefore, oxygen delivery, to the tumor, providing an effective way to reverse tumor hypoxia and tumor resistance to radiotherapy (155).

Modulating the cGAS-STING pathway offers another opportunity for cancer therapy, offering more specificity than cell-based approaches. In contrast to other pathways, the cGAS-STING signalling is not mediated by protein-protein interactions, but through the soluble second messenger cGAMP, synthesized by cGAS from ATP and GTP. It has been described that both chemotherapeutic agents (e.g. cisplatin and etoposide) and radiotherapy induce DNA damage, which can activate the cGAS-STING pathway promoting immune responses and enhancing tumor cell death (156, 157). Due to genome instability, some tumor cells spontaneously produce cGAMP (158). Moreover, cGAS-STING axis was essential for the antitumor effects of ICIs (159). In this regard, several cGAS-STING agonists, including STING-binding molecules and cGAMP derivates, have been developed, as apart from synergize with ICIs, they can enhance the antitumor effect of tumor vaccines, chemotherapy and radiotherapy (160).

However, STING activation can also promote tumor growth (161), metastasis (162) and enhance autoimmune diseases (51), suggesting that immunotherapies need to achieve a correct balance between stimulating an antitumor response and avoiding tumor-promoting inflammation. In this regard, small-molecule-based approaches have been developed to target cGAS-STING axis. cGAS inhibitors can be classified into catalytic site inhibitors, which bind to the active site and are competitive with ATP, GTP or cGAMP, or inhibitors that disrupt the DNA binding of cGAS, impeding the initiation of the signalling. STING antagonists on the other hand, can bind to the cyclic dinucleotide (CDN)-binding site, and therefore block STING activation, or can bind to cysteine residues near the transmembrane domain of STING, avoiding this way the palmitoylation and consequent activation of STING, as these residues are palmitoylated after CDN-stimulated activation (163).

The elevated glycolytic activity of solid tumor cells leads to an increase in lactate, protons and carbonic acid in the extracellular media, generating acidosis in the tumor microenvironment (164). Acidosis, like hypoxia, contributes to drug resistance (165) and immunosuppression (123). Therefore, four main therapeutic approaches have been developed to correct or avoid acidosis: 1) buffer therapy, consisting on administrating weak bases, 2) drugs selectively active at low pH, 3) agents that block cellular responses to acidosis, and 4) agents targeting the pH regulatory machinery itself (166). Most of the developed therapies against tumor acidosis target CAIX, which promotes the passive efflux of CO_2_ through the plasma membrane, and it is essentially only expressed in tumor cells (167). The monocarboxylate transporter family (MCT) members, which transport lactate through the plasma membrane, are potential targets to control tumor acidosis as well, however, MCT inhibitors are still at an early stage of development (168).

Another way of bypassing hypoxia-induced immunosuppression is targeting the adenosinergic signalling. Inflamed and cancerous tissue release ATP into the extracellular media, which is degraded into adenosine by CD39/CD73. Adenosine will eventually signal through A2AR receptor on immune cells and block their activation and activity. Silencing A2AR enhanced the efficacy of adoptive cell therapy (169), similarly, A2AR blockage improved CAR-T therapy (170) and addition of anti-PD-1 further ameliorated it (171). Preclinical models showed that combining A2AR antagonists with ICIs promoted tumor regression, moreover, clinical trials also showed that A2AR antagonist alone or in combination with ICIs resulted in better outcomes (172, 173). Similarly, small molecule inhibitors or monoclonal antibodies against CD39 and CD73 are being developed as potential anticancer therapies. Like A2AR blockage, anti-CD73 therapy improved ICIs therapy in various preclinical cancer models (174) and it showed a strong antitumor effect in clinical trials (175, 176). This is particularly relevant to target adenosine-rich tumors.

It will be critical to link these developments to biomarkers of expression of these targets, to classify patients for the relevant therapies. But expression alone is not necessarily enough depending on function-expression relationship. Finally, the marker to be useful, needs to relate to therapeutic benefit. Development of an accompanying diagnostic or theranostic will help bring these new strategies to fruition, in the selected patients.

## Discussion

Here we summarize hypoxic effect on tumor progression focusing on the immune system evasion, specifically we addressed hypoxic effects on the type I IFN pathway. Hypoxia generated immunosuppressive environment not only affects tumor cells, but also immune cells present in the tumor microenvironment which can be either recruited to support immune system evasion or inhibited to avoid their antitumor activity. Similarly, we have shown that molecules generated in a hypoxic environment, such as lactate or adenosine, can also contribute to this immune evasive phenotype, and that they are potential therapeutic targets. Moreover, targeting these molecules, as well as targeting hypoxia, could improve the efficacy of other therapies, e.g. immunotherapy. In this regard, an active type I IFN signalling is necessary for many anticancer therapies, however, the level of the IFN response needs to be controlled to avoid an excessive activation which could lead to detrimental effects. As with other signalling pathways, hypoxia regulates the type I IFN signalling and its effects would need to be considered when developing new therapeutic strategies.

## Author Contributions

EA and AH wrote the manuscript. All authors contributed to the article and approved the submitted version.

## Conflict of Interest

The authors declare that the research was conducted in the absence of any commercial or financial relationships that could be construed as a potential conflict of interest.

## Publisher’s Note

All claims expressed in this article are solely those of the authors and do not necessarily represent those of their affiliated organizations, or those of the publisher, the editors and the reviewers. Any product that may be evaluated in this article, or claim that may be made by its manufacturer, is not guaranteed or endorsed by the publisher.
